# Second campaign of microclimate monitoring in the carcer tullianum: temporal and spatial correlation and gradients evidenced by multivariate analysis

**DOI:** 10.1186/1752-153X-6-104

**Published:** 2012-09-18

**Authors:** Giovanni Visco, Susanne Heidi Plattner, Patrizia Fortini, Maria Pia Sammartino

**Affiliations:** 1 Rome University, La Sapienza, Math. Phy. Nat. Science Faculty, Piazzale A.Moro 5, 00185, Rome, Italy; 2 Cultural Heritage Superintendence of Rome City Council, Pz. Lovatelli 35, 00185, Rome, Italy

## Abstract

**Background:**

This paper discusses results obtained in the second monitoring campaign of the Carcer Tullianum, a particular hypogeum environment located in the historical centre of Rome (Italy). In the first paper we stressed the need to apply chemometric tools to this kind of studies in order to obtain full and significant information; really information on sampling design, sensors (type, number, position) and instrument validation seems to be not easy to find in literature for researches dealing with monitoring of indoor environments.

Also in this case three main parameters (temperature, humidity, illuminance) were monitored in the complex construction by an inexpensive self-assembled system along some horizontal and vertical vectors together with some measurements of oxygen, carbon dioxide and barometric pressure.

With respect to the first campaign, we used a higher number of sensors to cover a new excavated zone; for the same reason, as well as to take into account the presence of visitors, a different experimental design was adopted.

**Results:**

Different data treatments were applied to data coming from all the used sensors. A good view of the microclimate was obtained that also resulted coherent with the different position of the three rooms constituting the monitored site (Carcer, Tullianum, Convent). Classical time plots resulted useful to evidence the correlation of the main monitored parameters (T, RH% and illuminance) with macroclimate, as well as their delay in following macroclimate. Box-Whisker and Gain-Loss graphs evidenced at the best the microclimate differences between the three rooms; an almost hypogean microclimate was evidenced for the lower room (Tullianum) where humidity values range between 90 and 100% while lower values, but anyway higher than the external, and spread more widely were measured passing to Convent and Carcer with minimum values around 50% for the last. A scarce or very scarce correlation with macroclimate was evidenced for all the three main measured parameters. Lighting results mainly dependent on artificial light and only in few cases, but unfortunately in the most precious zone, illuminance exceeds values suggested by Normative.

**Conclusions:**

Box-Whisker and Gain-Loss graphs allowed us to have the best view of the microclimate for all the monitored rooms. The influence of lighting by lamps on the other monitored parameters resulted overlapped and clearly topped the solar one. The worst situation was found in the Carcer, where the presence of the main chandelier worsens the state of the frescoed walls, already subjected to wide changes in temperature and humidity. Also the lighthouse located above the Convent provokes lighting exceeding values suggested by Normative while, as expected, LEDs resulted as suitable source of light from a conservation point of view.

Susanne Heidi Plattner, Patrizia Fortini and Maria Pia Sammartino contributed equally to this work

## Background

For any material, microclimate changes are recognised as main responsible for speeding up their degradation. An indoor environment would reach an equilibrium that generally includes circadian cycles with, at different extent, smaller variation, similar frequency and a certain delay with respect to the outdoor climate (macroclimate). Many examples of how such equilibrium, as defined by Banham’s [1], must be considered the ideal conservation conditions for artefacts rely on the huge Cultural Heritage Patrimony that survived for hundred and hundred centuries even if in thermohygrometric condition that surely cannot be considered ideal (buried, dived). Unfortunately such equilibrium is incompatible with the enjoying of Cultural Heritage (CH) as all is needed to expose them to public, as well the visitors themselves, create environmental perturbation; so, a compromise must be reached to ensure the longest life of artefacts, as well as of the building containing them, that, in many cases as our one, can constitute the main CH. An accurate microclimate monitoring of such buildings must be the first step in a conservation project both to individuate the spontaneous condition and to design an eventual heating/cooling system (HVAC); the last must be set taking into account the CH safety and not the visitors comfort, this seems to be obvious but not always this rule is respected.

Microclimate never can be univocally defined in space while for a building a ‘mesoclimate’ can be defined, which is influenced by the building itself, its structure, geometry, shape and materials, as well as by the surroundings. Surely, macroclimate influences micro and mesoclimate and must be considered for a correct evaluation of the building insulation; for our purpose we define a macroclimate, characterised by locally measured meteor-climatic values, which are not influenced by the building itself [2].

In this paper we present results obtained in the monitoring of a particular hypogean environment that includes two underground levels of a wider building located in the historical centre of Rome. In a previous paper [3] we presented analogous results for the first monitoring campaign of the same site; in that occasion the site was closed to public and we had the opportunity to evaluate the trend of the microclimate toward the spontaneous one. We repeated the measurements exactly in the same period in order to evaluate the influence of visitors and of a new excavated room. Obviously, for the last, this must be considered the first monitoring campaign, so, we have no data of the trend toward its spontaneous microclimate. A comparison of data between the two monitoring campaigns will be treated in a next paper.

But what microclimate can be considered “a good conservation condition”? Italian normative rules on the control of microclimate through 5 UNI provisions [4-8] while up today the European Community only published three, treating the same topics of the UNI 11120, UNI10969 and UNI10829; the first deals with the measurement of the temperatures of the air and the surfaces of objects, the second gives guidelines for heating systems, while the third suggests some optimal value for conservation of inorganic hygroscopic materials [9-11]. Some ideal ranges of temperature and humidity for some materials including stone ones are suggested but maximum variation is given only for humidity. Unfortunately many papers on microclimate studies of indoor environments, especially in the field of cultural heritage conservation, could be discussed critically, because they do not state the type, number or position of the sensors used. Often the Design of Experiment (DoE), the result and instrument validation is not described. Sometimes only one sensor is used and, even if a short time is required (1 hour) to perform measurements in all the microenvironment, the control cards and/or univariate graphs used to treat data cannot be considered rigorous as it is obviously impossible to follow variation in time and space contemporarily [12].

As in the previous paper [3] we propose a full analytical procedure that foresees the preliminary and final control of the sensors accuracy and coherence, an experimental design that allows to cover at the best all the considered environment, a really too short monitoring period and a chemometric analysis of data. More, correlation between the monitored parameters (temperature, humidity and lighting) and macroclima data, kindly provided by the “Collegio romano” was looked for and some examples are reported from Additional file 1: Fig.0P to Fig.DA (all figures on external data file (EDF) have a capital letter as index).

## Experimental

The position of the building in the ancient Forum Romanum is shown as fig 0A in the EDF; it was probably built in the V or VI century B.C., above a water table.

**Figure 1:**
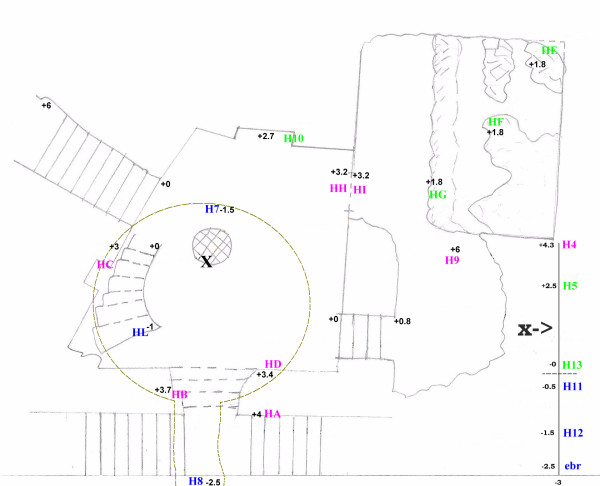
**Sensors position according to the Sampling Design, the black numbers indicates the quotes in meters with respect to the Carcer’s floor.** The blue labels group all sensors above the Carcer’s floor; the green and purple labels indicate the quote over the Carcer’s floor at an intermediate and higher level respectively.

Complete information on the buildings history can be found in a paper of D.B.Kerner [13] while it is possible to navigate the whole building on the web site maintained by the Roman Superintendence on Cultural Heritage (unfortunately up today only in Italian) [14]. Today the Carcer is a partially buried church accessible by a descending stair with about 20 steps while a relatively recent stair joints Carcer and Tullianum. In the previous campaign, the last had the floor continuously covered with a stratum of water rising from the underlying water table [15] while now the excess water is continuously pumped out. A new excavated zone dug out the foundations of a Convent that was also monitored. The entire complex is not equipped with a HVAC. In Figure 1 the position of the 21 used sensors with the quote respect to the Carcer’s floor while Fig.0B in the EDF give a better three-dimensional view.

Nineteen Hobo U12-012 by Onset USA were used to monitor humidity (RH%), temperature (°C) and illuminance (candles/ft^2^ or lux).

One Hobo U12-006 by Onset USA was equipped with a TMC20-HD sensor and was used to monitor the temperature of the spring water; it was inserted for about half meter in the junction of floor blocks to reach the water. In this paper it is named Hobo14.

One EB20-THP, by Ebro GmbH, Germany, equipped with certified sensors for humidity (RH%), temperature (°C) and air pressure (mbar) was used too.

Hobo calibration and coherence was controlled before and after the measurement campaign [3] using the above described Ebro and two certified Hg thermometers AA grade (VWR cod. 61027-056). Sensors were considered coherent when differences between them not exceeded the accuracy declared by the seller. No significant differences were found between values measured before and after the monitoring.

One LabQuest data station equipped with sensors for CO_2_, O_2_ and temperature measurements was used as well (CO_2_-bta, O_2_-bta and STS-bta by Vernier Tech., USA).

Meteorological (macroclimate) data was kindly provided by "Osservatorio Meteorologico of Collegio Romano", which is active since 1782 and located on the top of a 63m (above sea level) tower in the centre of ancient Rome, situated in 41.900°N, 12.480°E. Tullianum instead is situated in 41.893°N, 12.484°E.

Data elaboration softwares were mainly free ones, e.g. Past [16], Datalab, Gnumeric, WinIDAMS, Libre Office and some commercial ones like MVSP and Lotus 123.

To read data from data-loggers producer softwares were used (available on brand web site). Only one software was not free and we apologize for this.

## Methods

Twenty-one data loggers were employed as passive samplers to monitor the three parameters that mainly influence the conservation state of an object, temperature (°C), relative humidity (RH%) and illuminance (lux); thanks to their small dimension, they were easily fixed along vertical vectors with no significant interference on the spontaneous environmental air flux as well as without inconvenience from and toward visitors.

As in the previous, in this campaign CO_2_ and O_2_ were also monitored.

When passive samplers are employed sampling frequency must be accurately selected, in this case we use a sampling frequency of 5 minutes.

### Sampling design

Due to the presence of visitors, it was impossible to put all sensors in the identical positions of the previous campaign; anyway the experimental design was almost similar and base on one main vertical vector from bottom to top and some horizontal ones. Five sensors were used to cover the new excavated zone that will be called Convent in the following.

As in the previous campaign 6 sensors (Ebro, H12, H11, H13, H5 and H4, Figure 1) constitute the main vertical vector, it starts from the bottom of the central part of the Tullianum and, crossing the connecting grid, end to the top of the Carcer. An horizontal vector (H7, H12, H8, in Figure 1) starts from the altar inside Tullianum, crosses the vertical vector on the second point and ends inside the tunnel going toward the Cloaca Maxima. One vector starts from the main entrance of the Carcer, crosses the vertical vector at the fifth point from the bottom and ends on altar (horizontal vector, HB, H5 and H10, Figure 1). Four sensors (HI, HG, HF, HE Figure 1) are put in the Convent on a horizontal vector, it starts in the bordering Carcer with the HH sensor that is located near the glass wall dividing the two rooms. The remaining sensors were put as follows (Figure 1): one on the stair connecting Carcer and Tullianum (HL), one on each of the two frescoed walls (HC and HD), one on a catwalk overlooking the Convent (H9) while the last was put on the external part of the main entrance (HA). Sensors for oxygen and carbon dioxide were very close to Ebro just on the lower position of the main vertical vector. The sensor H14 was used to measure the temperature of the water tablet underlying Tullianum.

### Sensors calibration

As above said, just before and after the measurement campaign, all sensors were checked to control their rising time, accuracy, hysteresis, and coherence. This allows to estimate the sensors response to environmental variations, e.g. a coherence of 0.5°C allows us to regard a variation of 0.8°C between two sensors as significant; a more detailed procedure is described in our previous paper [3].

### Data treatment

Data obtained by all sensors was put in a 3D-matrix composed by 21 columns, with position points as variables, 6315 rows for each sampling time and 7 layers for temperature, RH, illuminance, barometric pressure, rainfall, CO_2_, O_2_. Some layer columns are empty as not all data loggers can detect all parameters. For example the barometric pressure layer has only two filled columns (Ebro sensor and meteorological data).

The above described matrix was then studied by Exploratory Data Analysis in order to identify any correlation between macro and microclimate as well as to identify the rooms spontaneous values.

Data of each measured parameter (layer) was plotted in monovariate graphs. Normally Run-Sequence plots of parameters (temperature, humidity, light intensity.) versus time or a function of time (here minutes from midnight of the first day 18th April).

In these graphs prefixed limits can be added and Shewhart Charts are obtained (also known as Control Charts), which enhance the environments characteristics.

The successive step in EDA is data analysis by Box & Whisker Notched Box Plots of a single parameter (a matrix layer), where variables (sensors) are plotted on the X-axis and raw measured distribution on the Y-axis.

This powerful analytical tool shows, here in the Massart [17] version, in the median domain, the distribution, spread, skew and percentile values 5% and 95% by whiskers; no outliers are shown. In our case only one matrix layer was plotted at a time so avoiding problems which can arise when variables with different scaling are plotted together.

Successively five Scatter Plot Matrixes (SpM) were designed showing up all possible Var-Var graphs between all variables (sensors), starting from raw data, in order to enhance correlations as well as distribution type of each variable on the diagonal. In Additional file 1: Fig. 0 F the SpM of all sensor is shown, in Additional file 1: Fig. 0 G the SpM among measured gas and macroclimate is shown. To better view the Additional file 1: Figs. 0 H, 0I showing respectively the SpM of RH% vs lux, the temperature vs lux, and finally the Additional file 1: Fig. 0 J shows the SpM of temperature vs RH%. On the diagonal of the Scatter Plot Matrix a histogram of ten bars is shown for each variable, which roughly allows identification of the distribution form; the values on histogram show the height of the major bar as the total is normalised to 1.

This important graph is often poorly substituted by a correlation matrix, using the r^2^ Pearson coefficient; this latter works only on linear data, which is not always the case, especially in the cultural heritage sector.

A building’s isolation can be evaluated also by Loss/Gain for Transmission graphs, where internal and external data are shown and compared to a perfect correlation which is shown as graph diagonal. Any point above or below the diagonal represents a building’s condition, not exactly correlated with the outdoor environment, e.g., in a temperature graph presence of points above the diagonal, can be due to a heating system. By using labels for single data points these graphs allow also evaluation of hysteresis, spontaneous behaviour, changes with time. The sensors accuracy is fundamental in these graphs. Practically all methods to measure averages can be used in this graph, in our case we used the median and, only in one case, the geometric mean. Unlike the arithmetic mean, the geometric mean, tends to dampen the effect of very low or very high values, which bias the arithmetic mean if used in computation of “central values”.

A correlation study between the three main measured parameters was the next analysis step. Thus two columns of two overlaying layers were compared and for each point variation of both parameters as well as the 98% confidence ellipses were obtained. Most of such graphs are shown in the EDF in Additional file 1: Fig.0P, Fig.0 T, Fig.0X and so on.

Fourier transformer was used to evidence the periodicity and intensity of the parameters variations.

## Results

The huge amount of data collected during the monitoring campaign doesn’t allow us to describe all results in detail; raw data together with part of the data treatment are available in the EDF while along the paper only the most relevant results will be presented and discussed.

**Figure 2:**
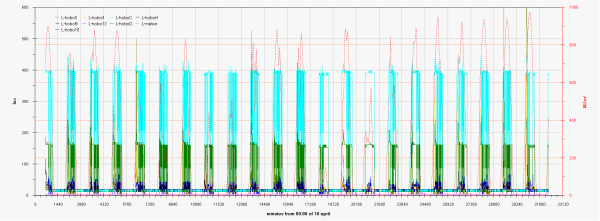
Trend of illuminance inside Carcer during the measurement campaign and comparison with external irradiation.

**Figure 3:**
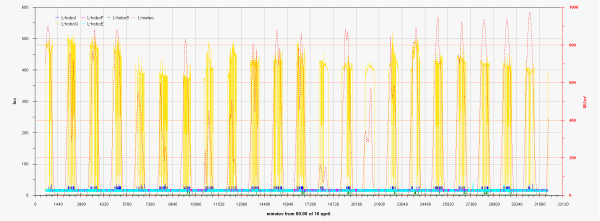
Trend of illuminance inside Convent during the measurement campaign and comparison with external irradiation.

**Figure 4:**
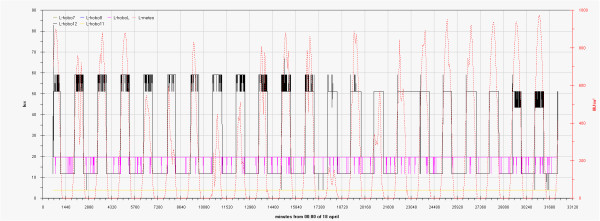
Trend of illuminance inside Tullianum during the measurement campaign and comparison with external irradiation.

**Figure 5:**
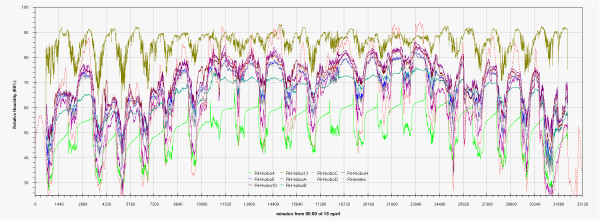
Trend of relative humidity inside Carcer during the measurement campaign and comparison with macroclimate.

**Figure 6:**
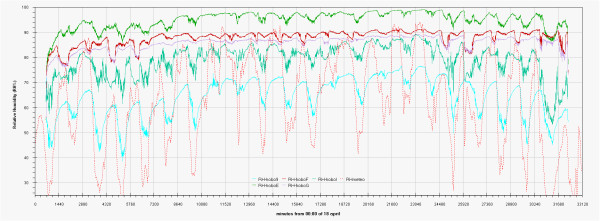
Trend of relative humidity inside Convent during the measurement campaign and comparison with macroclimate.

**Figure 7:**
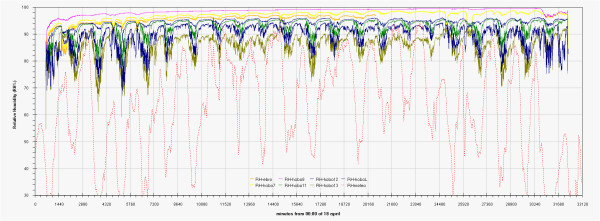
Trend of relative humidity inside Tullianum during the measurement campaign and comparison with macroclimate.

**Figure 8:**
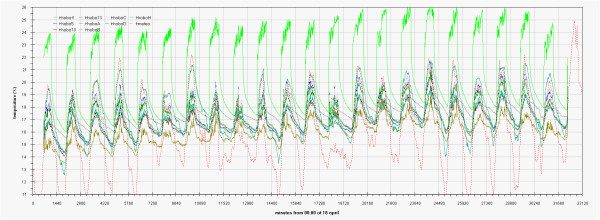
Trend of temperature inside Carcer during the measurement campaign and comparison with macroclimate.

**Figure 9:**
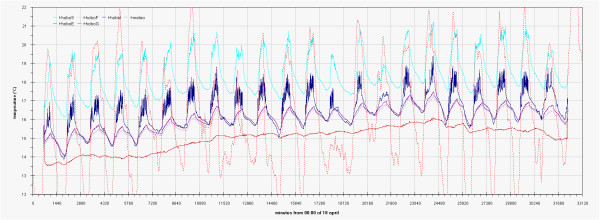
Trend of temperature inside Convent during the measurement campaign and comparison with macroclimate.

**Figure 10:**
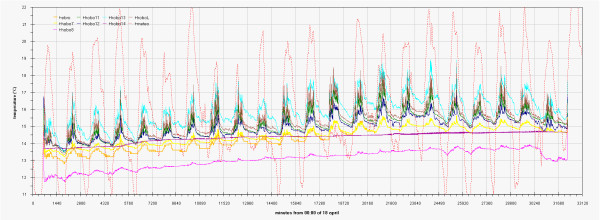
Trend of temperature inside Tullianum during the measurement campaign and comparison with macroclimate.

As can be seen in the EDF (Additional file 1: Figs 0C,0D,0E) the time plots of the three main monitored parameters (T, RH, Lux) are almost impossible to discuss if reported for all the sensors together, no significant improvement was attained by row or column centring. A better view can be obtained drawing time plots of each variable in each of the three different rooms (Figures 2, 3, 4, 5, 6, 7, 8, 9 and 10). In all Figures 2, 3, 4, 5, 6, 7, 8, 9 and 10, circadian cycles are evidenced that, with the exception of graphs reporting illuminance, are enough correlated with the macroclimate. Figures 5 and 8 show the expected trend along the vectors with maximum variations between the ends of the part of the vertical vector crossing the Carcer, as a fact they result very high, i.e. until 30% RH (that is 3 times higher than that suggested by normative for stone materials), and 6–8°C for Temperature; in all the other points differences are lower but anyway until 10% for RH and 3°C for Temperature.

Higher variations are evident in the Convent (Figures 6 and 9) where differences until 45% were registered between the sensor located on the catwalk and the one located close to the wall bordering the outside embankment; for the same sensors a maximum difference of 6°C was registered for Temperature. In such graphs the trends for the single sensor is almost flat for the Hobo E while daily cycles reach the maximum spread for Hobo 9, this clearly evidences the opposite effects of the high humidity coming from the outside embankment and the high heating provoked by the lighthouse located on the catwalk.

Inside Tullianum obviously we registered lower temperature and higher humidity with, in both cases, lower variations. For both variables, trends result enough flat with variations lower than 5% for RH and 0.5°C for temperature with the exception of the sensors more influenced by the Carcer microclimate, i.e. Hobo13 and HoboL that are respectively located on the grid and the stair connecting Carcer. The Loss-Gain and the Box-Whisker graphs resulted the best representation as let to summarise in a more easy to read form, all information contained in the time plot graphs. Taking into account that our data fit a non-normal distribution, in these graphs the median resulted the more robust estimator of the “central value”. The two types of graphs give complementary information as the Loss-Gain allows to a quick evaluation of the microclimate variation along all the monitoring period as well as of the influence of macroclimate while the Box-Whisker evidences the variation around the median and can be useful to evaluate the distribution shape for the single sensor calculated on all its measurements. In the Loss-Gain graph of illuminance we used the geometric mean as it resulted the most suitable to decrease the statistical weight of the big number of zero values measured during the night that let to a wrong evaluation of the real situation.

In the previous campaign, performed during a closing period of the site, almost all sensors showed no significant variation for light intensity, so related graphs were not considered; in this campaign, due to the presence of visitors, the situation was very different; so here we describe some useful details of the site. Sunlight only enters the Carcer to a very small extent as the unique entrance is under the street level and, more, the large external stair is protected by a roofing; as a consequence, the only Carcer’s wall directly exposed to sunlight is the one hosting the altar, just opposite to the entrance, which was monitored by Hobo 10; anyway Figure 2 shows that the illuminance measured in this point is enough low (about 50 Lux) during daylight. The Hobo 5, located on the vertical vector shows some values up to 200 lux but only for ½ hour at 10:00. Artificial lighting in the Carcer come from only one chandelier located in the central higher part of the ceiling. The Convent borders with Carcer and can be entered by some light from the above said chandelier through a glass window connecting the two rooms (1.90 m above the Carcer floor); it is also lighted by a LED raw crossing the room under the ceiling and from a lighthouse placed on a catwalk located in the upper level, the last is lighted by sunlight from its entrance and by a video system during the visits. Tullianum is lighted by a LED raw placed on a catwalk starting from the stair connecting Carcer and can receive some light from the Carcer’s chandelier through the grid connecting the two rooms located in the ceiling centre.

**Figure 11:**
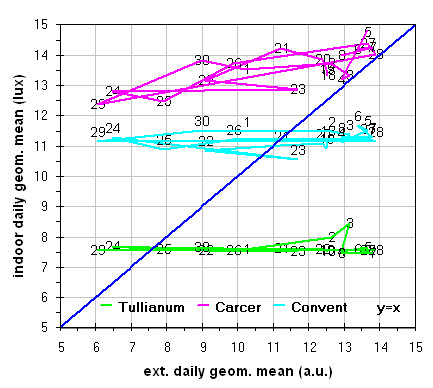
Gain-Loss graphs for illuminance measured in all the site with respect to outside.

The Loss-Gain graph in Figure 11, as expected, evidences inside Tullianum the lower values of illuminance that, with 2 exceptions are always lower than the external, such values result about 2 times lower with respect to the Carcer where the maximum values, always higher than the external ones, were measured; mid values were instead measured in the Convent. The flat or almost flat trend of illuminance in all the three rooms during the day evidences no correlation (Tullianum and Convent) or a very scarce correlation (Carcer) between the inner situation and sunlight; such results are coherent with what was said above, i.e. the Carcer is the only room directly exposed to sunlight but at almost scarce extent while the other two rooms are mainly lighted by lamps or Leds; so, as expected, the illuminance inside the site would be mainly imputed to the lamps.

**Figure 12:**
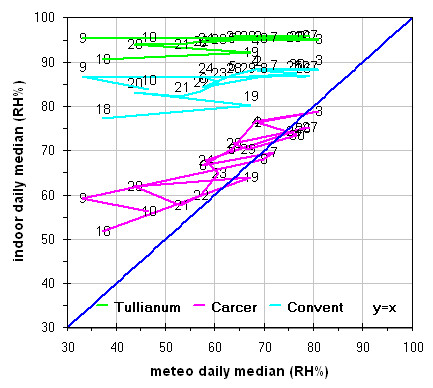
Gain-Loss graphs for relative humidity measured in all the site with respect to outside.

**Figure 13:**
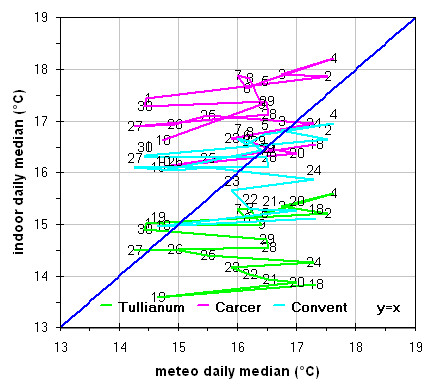
Gain-Loss graphs for temperature measured in all the site with respect to outside.

The analogous graph for humidity (Figure 12) shows well evident different trends in the Carcer with respect to the other two rooms for which in turn, even if not overlapped, trends are closer. Values measured by sensors located in each room follows the order Tullianum > Convent > Carcer while for the values spread the order is inverted i.e. Carcer > Convent > Tullianum so being coherent with their isolation (i.e. Tullianum at a lower level and practically not connected with outside, Carcer at higher level and directly connected with outdoor while Convent, even if at higher level with respect to Carcer, is not directly connected with outside), more all values are higher than the outdoor ones so evidencing the prevailing effect of the hypogean environment and of the water table underlying Tullianum. A sensor, connected to the datalogger Hobo14, was dipped in the spring present inside Tullianum to measure the water temperature. Figure 10 shows enough clearly that the temperature measured by all the other sensors have the same increasing trend of the water tablet. In the last Loss-Gain graph (Figure 13) the trend of temperature is reported. It can be seen that the trend inside the Convent overlaps the other two on the opposite sides while an analogous total variation during the entire monitoring period is evident for all the three groups. With some exceptions, values are higher and lower, with respect to macroclimate, for Carcer and Tullianum respectively while the Convent trend is almost cut in half by the theoretical correlation line.

**Figure 14:**
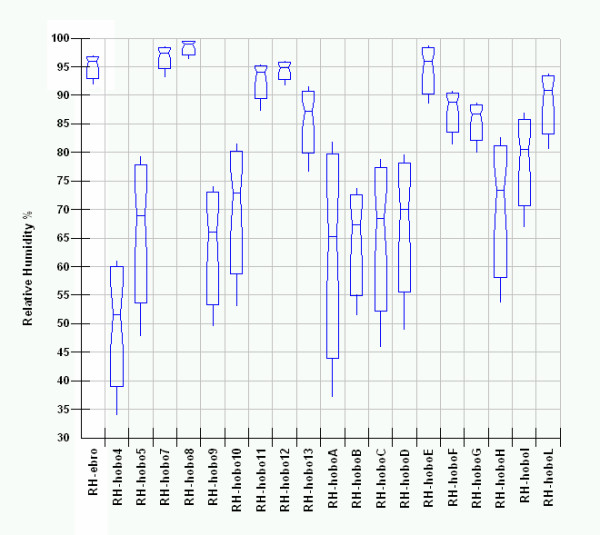
Box&Whisker plots for relative humidity measured by all the sensors.

The Box-Whisker graph in Figure 14 summarises the humidity data for all the sensors. The Hobo A, located outside, just on the main entrance, shows the higher spread, it can be used as mesoclimate reference. It is enough evident that values measured by all the other sensors are folded in two groups that significantly differ in spread. The first, showing lower variation, groups values measured in the most isolated points i.e. the vector in the Convent, the lower part of the inner stair and all points inside the Tullianum; in the last, excluding measurements from H13 that is located just under the grid connecting Carcer, for all the other points (measures by ebro, H7, H8, H11, H12), a smaller well separated group shows the highest mean values (94-99%) and the lowest spread (<6%).

For the second group of points higher spread and lower median values can be evidenced.

Some trends can also be seen along vectors. The expected decreasing trend, from the bottom to the top, is evident along the main vertical vector while along the main horizontal one an increasing trend can be seen starting from the main entrance (HoboB) and ending in the inner part of the Convent (HE); a lower spread and a higher difference of the median values between the ends can be clearly seen in the last room. The median humidity values measured by the two sensors located on the opposite part of the glass wall differ similarly to those of the Convent while the spread increases significantly passing to the Carcer. The two sensors located on the frescoed walls show no significant difference with median values very close to outdoor. The sensor located on the catwalk, shows the humidity value nearest to the outdoor one, this is surely due to the airflow entering from the entrance of the upper level. The highest sensor (Hobo 4) is the only one showing a humidity lower than the external; the difference is enough great (about 14%) and clearly evidence the influence of the underlying chandelier.

**Figure 15:**
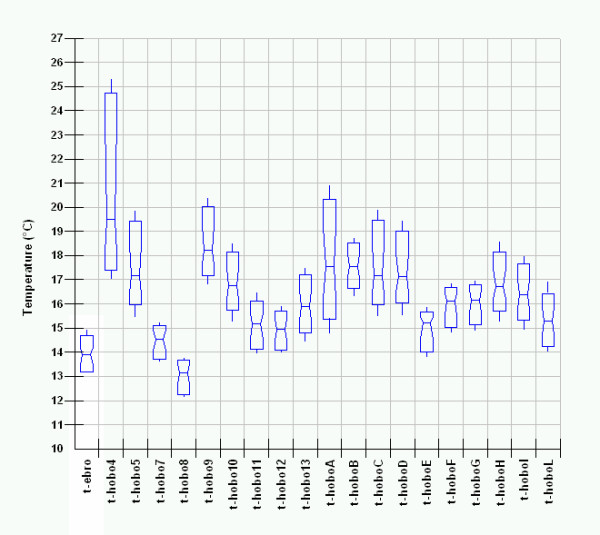
Box&Whisker plots for temperature measured by all the sensors.

Similar but opposite trends can be found in Figure 15 where temperature data of all sensors are reported.

Temperature measured by Hobo 4, ranges from about 17 to about 25°C with both median value and spread significantly wider respect to the external ones. Median value obtained for Hobo 9 also results higher with respect to the external one but with smaller extent (<1°C).

**Figure 16:**
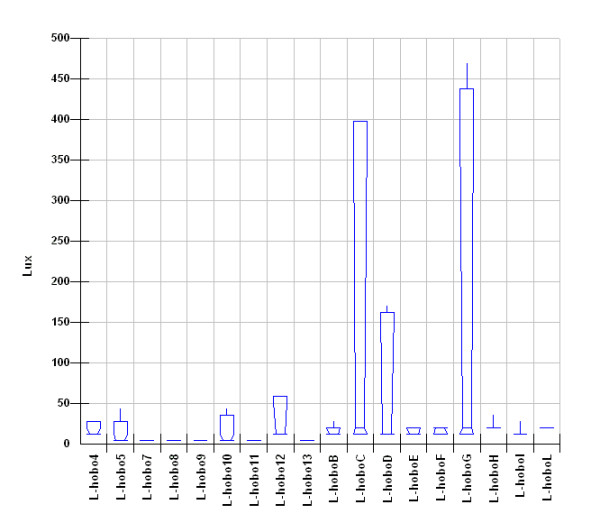
Box&Whisker plots for illuminance measured by all the sensors.

**Figure 17:**
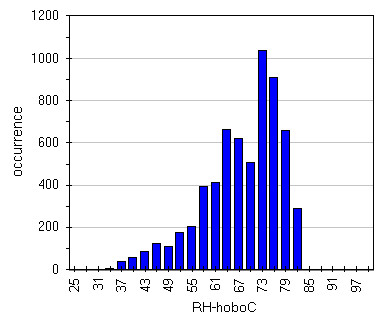
Distribution of humidity data measured by HoboC located on the most lighted frescoe.

**Figure 18:**
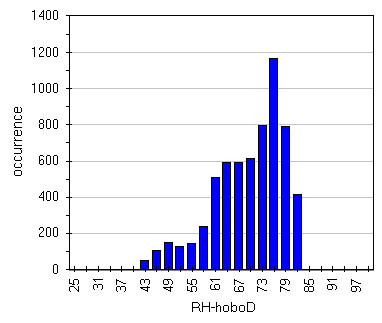
Distribution of humidity data measured by HoboD located on the less lighted frescoe.

**Figure 19:**
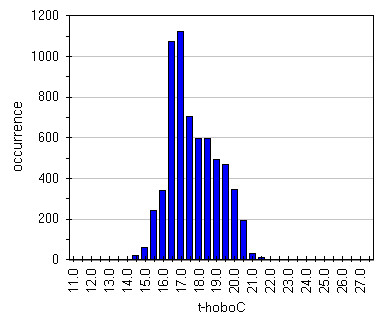
Distribution of temperature data measured by HoboC located on the most lighted frescoe.

**Figure 20:**
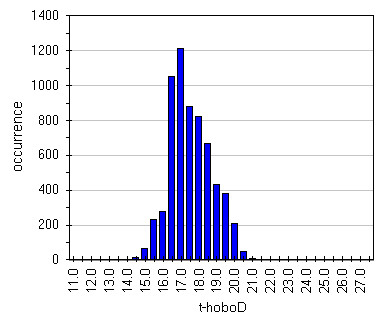
Distribution of temperature data measured by HoboD located on the less lighted frescoe.

Box-Whisker graph of illuminance (Figure 16) evidences the effect of lamps. It can be seen that, apart Hobo G, frescoed walls are the most lighted zones with a very wide spread that unfortunately means wide variation that in turn provoke humidity and temperature variations; really the last result 40–50% RH and 6.5-7.0°C (Figures 17, 18, 19 and 20). Lastly, it can be seen that, in most of the cases, the asymmetric distribution shapes evidence a higher frequency of higher and lower values for temperature and relative humidity respectively; this means that the site tries to reach spontaneous hypogean conditions.

Unfortunately all lamps were on in the time slot corresponding to the daylight so making difficult the separation of the two contributions on the single points. Anyway, in all the three graphs in Figures 2, 3, 4 the measured illuminance results independent from the sunlight while the enough regular cyclic trends surely correspond to switching on-off the lamps; this is coherent with what we said above, i.e. that lamps are almost the only source of light inside the site. Inside Tullianum, lighted by LEDs, illuminance is very low everywhere Figure 4. The failure of one or more LEDs can be noted; some broke definitively down after 9 days, others later (probably due to the unsafe environment with about 95% of RH%). It is also evident that LEDs remain on during the night between days sixteen seventeen.

In the zone of Convent lighted by LEDs illuminance is very low while the highest illuminance values in all the site were measured by Hobo G that is lit by light from the enough powerful lighthouse located on the catwalk and in many cases results more than 3 times higher than the one suggested by normative. It also can be noted that, with respect to the other two rooms, the lighting cycles are less regular (Figure 3), this is obviously more evident for Hobo G and could be imputed to the above listed multiple sources of light that discontinuously sum their effects to the one of the lighthouse.

As said above, even if the Carcer is directly connected to the outside, daylight scarcely enters the room, so measured illuminance must be mainly imputed to the chandelier. It is located in the centre of the room but the four equipping spotlights point toward different positions with different angulations. It is well evident that two of them point toward the frescoes which are the most lighted zones. In between the two frescoes a significant difference is also evident that can be imputed to a different distance and angulation of the spotlights; the following higher illuminance is measured by the Hobo 10 located on the altar that, as said above, is the only zone lit by daylight (Figure 2). It must be pointed out that the suggested illuminance value for frescoes is 150 lux that corresponds to about 14 foot-candles; even if the median value for the two frescoes are low, the maximum values are almost 2 times higher than the optimal one (Hobo C and D in Figure 16). Figure 2 shows that for the frescoes monitored by Hobo C the illuminance is about 2.6 times higher than the optimal one during all the period of lighting by lamp while a better situation is evident for the other frescoes as only some peaks exceeding the optimal value are evidenced.

As frescoes can be considered the most precious part of the site we here report other graphs showing that the lamp lighting can provoke an adverse influence on their thermohygrometric conditions, see Figures 17, 18, 19, 20. We have to stress that the influence of daylight on the lighting is very scarce in the Carcer but anyway its influence on the microclimatic condition is significant as influencing macroclimate that, in turn influences microclimate.

**Figure 21:**
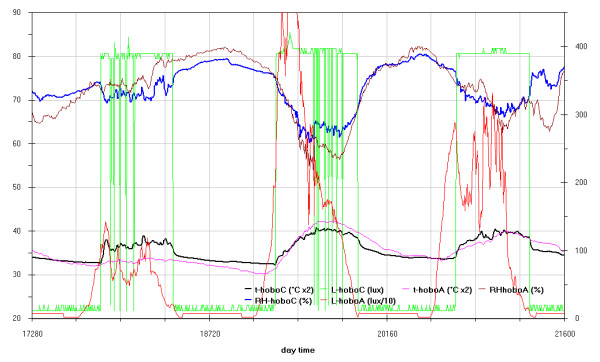
Comparison of data measured by HoboC with those by HoboA, located outside.

Using two Y axes and some linear scaling in Figure 21 a time plot is shown for illuminance, temperature and humidity measured by Hobo C and Hobo A; the latter is used, looking for correlation, instead of the meteo data as, in our opinion, it may be more representative of the real situation and anyway follows its trend. We chose three days where the first one was the most clouded one during the monitoring campaign; this lucky occasion gives us the possibility to have the best separation of the contribute of the lamp from that of sunlight on microclimate. The trends of Hobo C and Hobo A look different in the first day with respect to the other two; when the lamp is switched on, the humidity of the monitored point, compared to the external one, decreases with a higher slope and then maintains lower values until the lamp is switched off, then causing a quicker increase in humidity.

**Figure 22:**
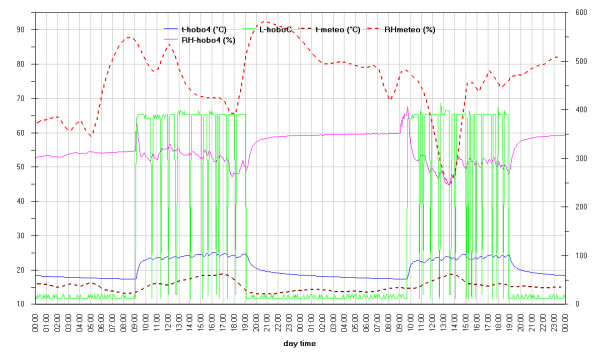
Comparison of the effect of chandelier (measured by Hobo C) and of meteo on the thermohygrometric conditions of the highest point inside Carcer (monitored by Hobo 4).

A further example of how the influence of lamps exceeds the one of sunlight is shown in Figure 22; Hobo 4 is on the Carcer top, so both for the normal air stratification and overall for its position just above the chandelier, we measured the highest temperature and low humidity, we also reported in Figure 22 the illuminance measured by Hobo C as representative of the lamp power. It can be seen that during the night the external humidity greatly varies while the inner one have an enough flat trend with only a slight increase. A marked increase seems to correspond to the lamp switching on but really corresponds to the opening of the main door; this increase occurs at around 9 o’clock each day while the artificial lighting not always starts immediately after; as an example, in the first of the two days considered in Figure 22 the door was open and lamp immediately switched on while on the second day, more sunny (see Figure 2 from time 10080 to 12960), lighting was delayed for half an hour. The following quick decrease corresponds to the lighting as well as the marked increase at the end of the lighting time corresponds to the lamp switching off. During lighting small variations correspond to each switching on-off of lamps probably matching any single visit. The trend of the temperature obviously gives the same information.

It is well known that degradation of any material depends mainly on the frequency of the thermohygrometric variations. In the above given example we have seen that we had both slow and small quick variations but also wide quick variations (on the starting and ending of lighting); the latter, for humidity, are close and in some case higher than the value suggested for stone materials by the UNI 10829 normative (10%, no data is given for temperature).

**Figure 23:**
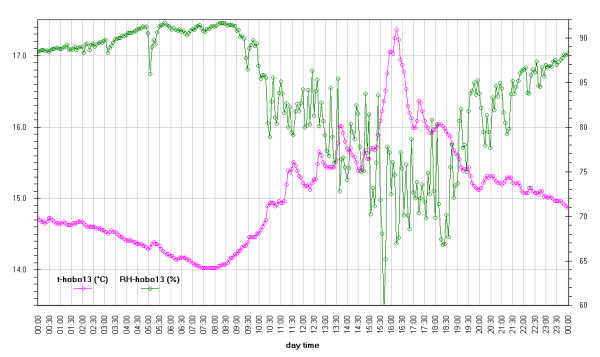
Daily variation monitored by Hobo 13 located just above the grid connecting Carcer and Tullianum.

Hobo 13 is located inside Tullianum but just above the grid connecting Carcer (really in all the graphs, we put it in both the rooms). Together with the inner stair the grid is the only point where an indirect exchange with outside is possible and, as a fact, Figure 10 evidences the greatest and quickest variation in such zones. Figure 23 gives the best view of such variations that in the worst case results of about 24% in 20 min for humidity and near to 2°C for temperature in less than 1 hour.

**Figure 24:**
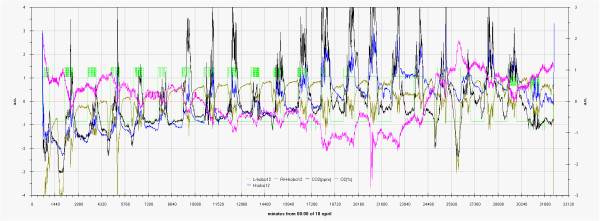
Influence of illuminance and thermohygrometric condition on the trend of oxygen and carbon dioxide inside Tullianum. Values were previously autoscaled.

In Figure 24 the trend of oxygen and carbon dioxide, measured inside Tullianum is reported together with temperature and illuminance measured by Hobo12, illuminance, also in this case, was used as indicator of the visitors presence. The data was pre-treated with autoscaling to change the point of view [18]. The expected opposite trend results for the two monitored gases while also in this case it is not easy to find a correlation between them and the light for the above said reasons. We had an help by the distraction of the site keeper that from 09:00 on 3rd May (Figure 4) forgot to switch off the lamps (23040 corresponds to midnight); it is clear that in this occasion temperature decreases to a lower extent during the night while oxygen and carbon dioxide followed the same trend as in the other days so demonstrating the prevailing effect of the visitors respiration with respect to the temperature.

Discrete Fourier Transformer was calculated with Past software but as shown in Additional file 1: Fig.0 N the main peak is the 1440 minutes periodicity, On Fig.0O sensor n.11 shows more peaks but invaluable for the analysis. Principal Component Analysis, calculated with MVSP after column centring [18], show only, in loadings, some sensor with large contribution to the model as in Additional file 1: Fig.0 L for Humidity and Additional file 1: Additional file 1: Fig.0 M for illuminance.

## Conclusions

Data treatment by Box-Wiskers and Gain-Loss graphs demonstrated to be very useful to evidence the microclimate variations both between the three rooms and inside each of them. The magnitude and frequency of such variations was successfully evaluated by normal time-plot in each of the room and evidenced the dangerous effects of the chandelier lighting the Carcer and of the normal lamp located on the catwalk inside Convent; on the contrary LEDs demonstrated to be suitable from the conservation point of view. The effect of the chandelier is particularly dangerous for the frescoes; at least the spot-lights must be positioned better in order to decrease the damage, especially the one that lit the frescoes monitored by Hobo C-D. The lighthouse located inside the Convent should be also eliminated or substituted by a less energetic lamp.

Surely the two campaigns were too short and covered only a seasonal period; so, excluding the suggestions above on lamps, it would be hazardous to suggest interventions to optimize the microclimate.

In our two monitoring campaigns we aimed to evaluate the influence of microclimate on the conservation state of the site. In literature there are many studies showing that certain gases can have a noticeable adverse effect even at low concentrations on typical building interiors [19] as well as previsions on the effect of climate change on historic interiors [20]. Surely, adverse chemical reactions must be considered because chemical and physical effects cross each other as well as with biological ones [21,22]. The inspection of the site revealed no so evident presence of biological species. It is well known that acidic gases such as carbon, nitrogen and sulfur oxides, have an adverse influence on the conservation state of any object in particular for calcareous stones [23]. We have done just an attempt to evaluate the effect of visitors as producers of carbon dioxide and consumers of oxygen through their respiration. In our two campaigns we only monitored this gases in the most isolated zone of the site because there we expected the greater variations, but surely an enough higher number of such sensors is needed to obtain useful information; on the contrary, the number of sensors for temperature, humidity and illuminance, even if it could be increased a little, resulted to be sufficient. Monitoring of other acidic gases will be needed as well as that of particulate, especially near the frescoes.

Finally all these data exploration methods together allow evaluation of the building’s isolation from the outdoor environment, as well as enhancing internal gradients; further spontaneous values, not normal behaviour and sudden changes, which are the most dangerous events for a cultural heritage site, can be evaluated.

## Competing interests

None of the authors received any financing, scholarship, contract or consulting from societies mentioned in this paper. Instrument were bought regularly through national research funding.

## Authors' contributions

Among the authors, which contributed in same manner to this work, we would like to differentiate SHP for precious work on English Language and coherence revision of the paper; further PF is responsible for the cultural heritage site for the Roman City Council, who contributed with information on the historic/artistic aspect. The remaining authors contributed in different manner but at the same level to this work. All authors read and approved the final manuscript.

## Authors' information

GV, SHP, and MPS: Rome University, La Sapienza, Mat. Phy. Natural Science Faculty, Rome, Italy.

PF: Cultural Heritage Superintendence of Rome City Council, Pz. Lovatelli 35, Rome, Italy.

## Supplementary Material

Additional file 1 **The first of the two .XLS files (MSexcel 97/2000 version) contains the raw data obtained from every sensor and all statistic parameters calculated for every column of the main matrix.** In the second we calculated the minimum, the maximun, the time when they occurred, the median and the spread as 95–5 percentile. All other files are figures from 0A to DA in .PNG format cited in the text and with explicative filename. All files was compressed in a single .ZIP file that can be opened using the major free or open source software as 7zip, MSviewer 2003, LibreOffice, Smartsuite 9.x, IrfanView, with some OS. Click here for file
